# Virtual Learning in Kindergarten Through Grade 12 During the COVID-19 Pandemic and Chronic Absenteeism

**DOI:** 10.1001/jamanetworkopen.2024.29569

**Published:** 2024-08-21

**Authors:** William N. Evans, Kathryn Muchnick, Olivia Rosenlund

**Affiliations:** 1 Department of Economics, University of Notre Dame, Notre Dame, Indiana; 2 National Bureau of Economic Research, Cambridge, Massachusetts; 3 Abdul Latif Jameel Poverty Action Lab, Cambridge, Massachusetts

## Abstract

**Importance:**

Chronic absenteeism among kindergarten through grade 12 students has increased considerably after the COVID-19 pandemic.

**Objective:**

To examine the association between virtual learning during the 2020-2021 school year and chronic absenteeism during the 2021-2022 school year at the school district level.

**Design, Setting, and Participants:**

This cross-sectional study used a panel of 11 017 school districts throughout the US comprising kindergarten through grade 12 for the 2018-2019 and 2021-2022 school years.

**Exposures:**

The key covariates were the percentage of hybrid and virtual school days in the previous school year, with an assumption that these values in the 2018-2019 school year were zero.

**Main Outcome and Measures:**

Chronic absenteeism rates at the district level, which were regressed on the percentage of school days in a learning mode in the previous school year, demographic characteristic and socioeconomic status controls, plus district and year fixed effects. Observations were weighted by district enrollment, and SEs were clustered at the district level.

**Results:**

The dataset includes 11 017 school districts for 2 years and 22 034 observations. Chronic absenteeism rates increased by 13.5 percentage points, from a mean (SD) of 15.9% (8.1%) in the 2018-2019 school year to 29.4% (13.2%) in the 2021-2022 school year. Students whose schools had 100% virtual instruction during the COVID-19 pandemic had chronic absenteeism rates that were 6.9 percentage points (95% CI, 4.8-8.9 percentage points) higher than those that were 100% in person. Hybrid instruction was not associated with increased absenteeism. The association between virtual learning and chronic absenteeism varied by socioeconomic status, with the conditional correlation much larger for at-risk students; chronic absenteeism rates were 10.6 percentage points (95% CI, 7.2-14.1 percentage points) higher among students with 100% of days in virtual learning from districts in the top quintile of poverty rates compared with 100% in-persion districts.

**Conclusions and Relevance:**

In this cross-sectional study, chronic absenteeism rates were substantially higher in school districts that used virtual learning during the COVID-19 pandemic compared with in person. Understanding how to reduce chronic absenteeism and use virtual learning without potentially negative consequences are key policy questions moving forward.

## Key Points

**Question:**

What is the association between the use of virtual learning in kindergarten through grade 12 education during the 2020-2021 school year and chronic absenteeism?

**Findings:**

In this cross-sectional study, data from 11 017 school districts from the 2018-2019 and 2021-2022 school years within a difference-in-difference framework show that districts with more virtual school days in 2020-2021 had higher rates of chronic absenteeism during the 2021-2022 school year. These higher rates are associated with results in districts with high poverty levels.

**Meaning:**

Key future questions include understanding whether this result is causal and why lower district income was associated with worse outcome.

## Teaser

This cross-sectional study examines the association between virtual learning in kindergarten through grade 12 (K-12) education during the 2020-2021 school year and chronic absenteeism during the 2021-2022 school year at the school district level.

## Introduction

Recent research has demonstrated that between the 2018-2019 and 2021-2022 school years, nationwide chronic absenteeism rates in kindergarten through grade 12 (K-12) education increased by 13.5 percentage points, a 91% increase overall.^1^ Chronic absenteeism has been associated with several negative outcomes, including lower test scores,^2,3,4^ a reduction in educational and social engagement,^2^ lower rates of high school completion,^4,5^ and higher rates of substance use.^6^ Understanding the factors associated with absenteeism is an essential step toward fostering students’ educational development and general well-being.

The increase in chronic absenteeism occurred as US public schools were returning to in-person instruction after the COVID-19 pandemic.^1^ School districts’ reliance on virtual and hybrid learning during the 2020-2021 school year raises the question of whether learning mode was associated with absenteeism rates. Rates of in-person instruction varied considerably along demographic, social, and political lines^7,8^ but were not correlated with disease incidence.^9^ There is a growing body of literature suggesting that the movement away from in-person instruction during the 2020-2021 school year reduced student achievement,^8,10,11,12^ worsened children’s mental health,^13,14,15,16^ and decreased school enrollment.^17,18^ Cross-tabulations from the Return to Learn Tracker web page indicate that there was little variation in chronic absenteeism rates at the district level in the 2018-2019 school year based on eventual in-person instruction rates in the 2020-2021 school year.^19^ However, absenteeism rates were substantially higher in districts in the 2021-2022 school year that were mostly remote compared with those that were mostly in person during the 2020-2021 school year.

In this study, we examine this issue in a more systematic fashion. We construct a panel dataset of 11 017 school districts for the 2018-2019 and 2021-2022 school years and examine whether the fraction of school days spent in hybrid or virtual instruction during the pandemic was associated with chronic attendance rates after the pandemic. Because districts did not provide virtual instruction in 2018-2019, the panel nature of the model can be thought of as a difference-in-difference model. Given multiple observations per district, we can control for the permanent, systematic differences in chronic absenteeism rates across districts.

## Methods

### Data Source

In this cross-sectional study, we constructed a district-level dataset that measured chronic absenteeism rates, student demographic characteristics, and characteristics of the population living within the district boundaries during the 2018-2019 and 2021-2022 school years. The data are outlined in more detail in eMethods 1 in Supplement 1 and reported briefly here. All the data for this project were from publicly available sources, and the data were aggregated to the school district level; as a result, this study was not considered human participant research per the Common Rule. Because this study was not considered human participant research, we did not obtain a waiver from the institutional review board. We followed the Strengthening the Reporting of Observational Studies in Epidemiology (STROBE) reporting guideline for cross-sectional studies.

Counts of students who are chronically absent by district or local education agency (LEA) are reported to the National Center for Education Statistics each school year. A student is defined as chronically absent if they miss at least 10% of instructional days in a given school year. To calculate absenteeism rates, we divided the number of students who were chronically absent by the total number of students in the LEA, available from the Common Core of Data. The Common Core of Data also reports counts of students’ self-reports of race and ethnicity. We used these values to calculate the percentage of students who were Asian non-Hispanic, Black non-Hispanic, Hispanic, White non-Hispanic, and other race non-Hispanic in each district. Other race included American Indian or Alaska Native, Native Hawaiian or Other Pacific Islander, 2 or more races, or race not specified. Race and ethnicity were assessed in this study because there are persistent differences in chronic absenteeism by race and ethnicity.

We collected several variables from the American Community Survey aggregated to the LEA level to use as controls. We used the 2019 5-year American Community Survey for the 2018-2019 school year and the 2022 5-year American Community Survey for the 2021-2022 school year. Finally, we merged information about school learning modes during the 2020-2021 school year from the COVID-19 School Data Hub, which reports the percentage of school days at the LEA level that were in person, hybrid, or virtual during the 2020-2021 school year. The web page does not have data from Iowa, Montana, or Oklahoma.

### Statistical Analysis

The basic statistical model exploits the fact that we have multiple observations per district. The model is outlined in detail in eMethods 2 in Supplement 1 and reported briefly here. Our balanced sample contains chronic absenteeism rates for school districts in 2 time periods: 2018-2019 (before COVID-19) and 2021-2022 (after COVID-19). We regressed these rates on the percentage of district school days in the previous year that were hybrid or virtual, assuming that 100% of school days were in person in 2018-2019. Control variables in the regression from the American Community Survey included the poverty rate (determined by the federal poverty level) and the real median income (in 2022 US$) for families with children younger than 18 years of age and the percentage of adults aged 25 years or older with a high school degree, some college, or a 4-year college degree (with the percentage having less than a high school degree being the reference group). Control variables from the Common Core of Data included the percentage of students who are Asian non-Hispanic, Black non-Hispanic, Hispanic, and other race non-Hispanic (with White non-Hispanic as the reference group). We also included a complete set of dummy variables for each district, plus a year fixed effect for the 2021-2022 school year. The district dummy variables controlled for permanent differences across districts, while the year fixed effect captured the time-varying effects common to all schools in a particular year but may vary over time (eg, the federal stimulus program, COVID-19). We weighted observations by district student enrollment each year, allowing for arbitrary correlation in errors at the district level.

The 2-way fixed-effects model can be thought of as a difference-in-difference estimator. Given that we have district and year effects, districts that moved to hybrid and virtual instruction were “treated” with differing intensities of alternative instruction over time. Within the difference-in-difference model, districts that had no hybrid or virtual instruction in the 2020-2021 school year were used as a comparison sample. Because these districts experienced no change in hybrid or virtual instruction over time, the time-series movements in chronic absenteeism represent the secular trend in this outcome that is common to all districts. The difference in these 2 differences is then an estimate of how absenteeism rates vary between virtual or hybrid districts and fully in-person districts.

In eMethods 2 in Supplement 1, we outline in detail why we selected the model we used and discuss some of its limitations. We considered statistical significance to be a 2-sided *P* < .05. All analyses were conducted using Stata/SE, version 16.0 (StataCorp LLC).

## Results

### Descriptive Statistics

Merging data from all sources using the National Center for Education Statistics LEA ID, we produced an analysis sample of 11 017 LEAs for which we had 2 years’ worth of data each, for a total of 22 034 observations. We refer to this dataset as our balanced panel of districts. This dataset represents roughly 87% of all K-12 students in the US in the 2018-2019 school year.

Table 1 reports basic descriptive statistics for the variables in our balanced panel of schools by year. We report values for key variables weighted by district enrollment for the year. Between the 2 school years, chronic absenteeism rates increased by 13.5 percentage points, from a mean (SD) of 15.9% (8.1%) to 29.4% (13.2%), which is identical to the numbers referenced in the Introduction.^1^ During the 2020-2021 school year, a mean (SD) of 39.3% (41.6%) of class days were in person, 33.9% (33.6%) were hybrid, and 26.8% (31.2%) were virtual. For the 2018-2019 school year, we assigned 100% of classes to be in person. We observed modest increases in the percentage of Hispanic students, Asian non-Hispanic student, and students of other races and ethnicities, as well as a slight decrease in the percentage of Black non-Hispanic students. In addition, there were slight decreases in the percentage of district population living in poverty and increases in real mean household income and the percentage of adults with a college degree living in district boundaries. As none of the demographic variables changed substantially over the 3-year time span, these characteristics will not explain much of the movement in chronic absenteeism over time.

**Table 1.** Descriptive Characteristics of Included Schools^a^

Variable	Sample mean (SD)	
	2018-2019 School year	2021-2022 School year
Chronically absent, %	15.9 (8.1)	29.4 (13.2)
Days in-person classes during previous school year, %	100.0	39.3 (41.6)
Days hybrid classes during previous school year, %	0	33.9 (33.6)
Days virtual during previous school year, %	0	26.8 (31.2)
Asian, non-Hispanic students, %	5.5 (8.1)	5.6 (8.4)
Black, non-Hispanic students, %	14.7 (17.6)	14.5 (17.2)
Hispanic students, %	27.3 (24.7)	28.5 (24.5)
Other race, non-Hispanic students, %^b^	5.1 (5.8)	5.8 (6.1)
District population in poverty, %	13.1 (6.8)	12.2 (6.2)
Adults in district aged ≥25 y with		
A high school degree, %	26.8 (8.4)	26.3 (8.6)
Some college, %	29.1 (5.9)	28.7 (5.9)
A bachelor’s degree or more, %	31.7 (14.6)	33.8 (15.0)
Children in district who live in a single-parent household, %	29.9 (11.8)	29.7 (11.9)
Median household income among households with children <18 y, real 2022, $	$90 719 ($40 687)	$96 119 ($41 044)
Districts, No.	11 017	

^a^ Descriptive statistics are weighted by district enrollment for the year.

^b^ Other race includes American Indian or Alaska Native, Native Hawaiian or Other Pacific Islander, 2 or more races, or race not specified.

**Figure 1.**
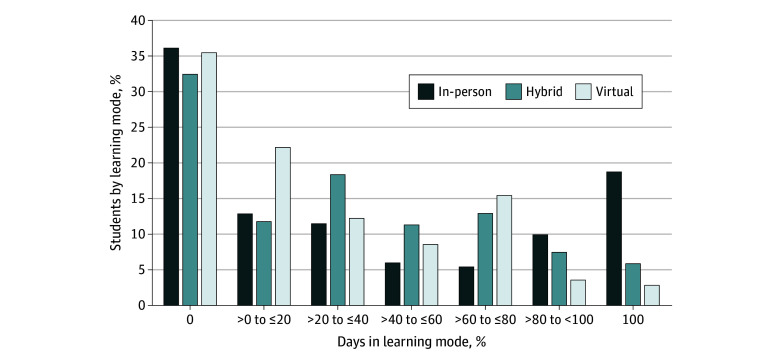
Share of Students by Learning Model in 2020-2021 School Year The values are weighted by district size in the 2021-2022 school year.

Figure 1 reports the percentage of students from our balanced panel that had a specific value of days in a learning mode during the 2020-2021 school year. We report data for 7 categories (0%, >0% to ≤20%, >20% to ≤40%, >40% to ≤60%, >60% to ≤80%, >80% to <100%, and 100%). The fractions sum to 100% within each mode; 36.0% of students had zero school days in person in 2020-2021, but 18.7% had 100% of school days in person. Approximately 22% of students had 60% or more school days held virtually during the 2020-2021 school year.

### Statistical Model Results

The results from our fixed-effects model are reported in Table 2. As much of the differences in absenteeism rates are between districts and not within districts over time, the *R*^2^ for the regression is high. These results suggest that students who spent 100% of school days in a hybrid or virtual setting during the 2020-2021 school year experienced a statistically significant increase in chronic absenteeism in the 2021-2022 school year (ie, 2.9 percentage point [95% CI, 1.6-4.3 percentage points]). This finding masks considerable heterogeneity in the effect based on learning mode; Table 2 also adds separate variables for percentage hybrid and percentage virtual days. Here, the coefficient on percentage of hybrid days is small, negative, and statistically insignificant (–0.3 percentage points [95% CI, –1.9 to 1.3 percentage points] for those with 100% virtual instruction). In contrast, the coefficient for percentage virtual days is large and statistically significant, indicating that students who spent 100% of their days in virtual schooling during the 2020-2021 school year experienced a 6.9–percentage point (95% CI, 4.8-8.9 percentage points) increase in chronic absenteeism during the 2021-2022 school year. With *P* < .001 for the model, we can easily reject the null hypothesis that the coefficients for hybrid and virtual variables are equal.

**Table 2.** Fixed-Effect Estimates, of Factors Associated With Chronic Absenteeism^a^

Covariate (hybrid + virtual)	Model 1	Model 2
% Days not in person, coefficient (95% CI)	0.029 (0.016 to 0.043)	NA
% Days hybrid, coefficient (95% CI)	NA	−0.003 (−0.019 to 0.013)
% Days virtual, coefficient (95% CI)	NA	0.069 (0.048 to 0.089)
Observations, No.	22 034	22 034
*R*^2^ value	0.89	0.89
*P* value, on test coefficient on days hybrid = days virtual	NA	<.001

Abbreviation: NA, not applicable.

^a^ Observations are weighted by total enrollment in the district. The 95% CIs were constructed allowing for arbitrary correlation in the errors within a district. Other covariates include district and year fixed effects; percentage of students who are Asian non-Hispanic, Black non-Hispanic, Hispanic, and other race non-Hispanic; percentage of people within the district boundaries in poverty; percentage of children in the district boundary living in single-parent households; percentage of adults aged 25 years or older within district boundaries with a high school degree, some college, or a 4-year college degree; and real median household income of households with children younger than 18 years within district boundaries.

Much of the variation in stay-at-home policies during the COVID-19 pendemic occurred at the state level. To examine whether we were capturing variation in state policy, we added a series of state-by-year effects to the model for the 2021-2022 school year; in this model, the result is unchanged in that the coefficient on virtual learning is 0.068 (95% CI, 0.047-0.089). In addition, the fundamental statistical association between basic demographic characteristics and chronic absenteeism could have been altered by the events surrounding the COVID-19 pandemic. Therefore, in a separate set of models, we allowed the coefficients on the control variables to vary in the 2021-2022 school year. These results are reported in eTable 1 in Supplement 1 and described in eMethods 3 in Supplement 1. Here, we added variables one at a time, then all at once. Adding these interactions separately did not significantly alter the coefficient on virtual learnings. Adding all these variables together still left a statistically significant positive coefficient on virtual learning.

One concern may be that the chronic absenteeism rates reflect differences in COVID-19 infection rates during the 2021-2022 school year, which could be due to differences in COVID-19 vaccination rates. As outlined in eMethods 1 in Supplement 1, we used population vaccination rate data at the county level as of the end of December 2021. We also calculated mean weekly COVID-19 per-person infection rates at the county level from August 1, 2021, through May 31, 2022. The infection data are for the entire county and do not measure infection rates for children. These 2 datasets are merged based on the county where the district is located. If the district spanned multiple counties, a simple mean was taken across all relevant counties. This merging reduced our sample to 10 812 school districts and 21 624 observations. When these 2 new variables (vaccination rate and infection rate) were added to the model, the coefficient on virtual learning is 0.068 (95% CI, 0.047-0.089), which is virtually identical to the results in Table 2.

Some have inquired whether the increase in chronic absenteeism for children receiving virtual instruction was due to post–COVID-19 condition symptoms. Most studies investigating this topic suggested that 10% to 20% of children exhibited post–COVID-19 condition symptoms.^20^ Post–COVID-19 condition would explain the positive coefficient on virtual learning only if COVID-19 incidence was higher in districts with higher amounts of virtual learning. Two factors argue against this. First, the Centers for Disease Control and Prevention estimate that 96% of children aged 6 months to 17 years had COVID-19 seroprevalence by the end of 2022; this percentage was 89% by March or April of 2022.^21^ Such high seroprevalence rates nationwide would suggest little variation across districts. Second, evidence suggests that aggregate COVID-19 infection rates are lower in areas with more virtual instruction.^22^

Previous work has shown that the degree of in-person instruction varied considerably by underlying characteristics of the district^7^; this pattern is present in our data. In Table 3, we report the share of each learning model in quintiles of poverty rates within district boundaries during the 2018-2019 school year using data from the American Community Survey. Districts with higher poverty rates had notably higher rates of virtual learning than districts with lower poverty rates. In contrast, the fraction of hybrid classrooms decreased appreciably from the area with the lowest to the highest poverty rates. Table 3 also reports the chronic absenteeism rates in the 2018-2019 school year. These results show a large increase in chronic absenteeism as poverty rates increase, suggesting that virtual instruction was used in districts that were more at risk for chronic absenteeism.

**Table 3.** Fixed-Effect Estimates of Factors Associated With Chronic Absenteeism by District-Level Poverty Rate in 2018-2019 School Year^a^

Variable	Quintile of district-level poverty rate, 2018**-**2019 school year				
	1	2	3	4	5
Days in mode school year 2020-2021, %					
In person	37.3	35.8	46.7	46.8	30.7
Hybrid	45.6	38.7	30.4	26.2	31.7
Virtual	17.0	25.5	22.8	27.0	37.6
Chronically absent during 2018-2019 school year, %	8.9	12.9	15.2	18.0	21.3
Regression parameter estimates (95% CI) on days in mode					
% Days hybrid	0.001 (−0.019 to 0.022)	0.008 (−0.017 to 0.034)	0.001 (−0.021 to 0.024)	−0.014 (−0.043 to 0.014)	0.017 (−0.011 to 0.045)
% Days virtual	−0.033 (−0.065 to −0.001)	0.027 (−0.016 to 0.071)	0.033 (−0.009 to 0.075)	0.086 (0.047 to 0.126)	0.106 (0.072 to 0.141)

^a^ Observations are weighted by total enrollment in the district. The 95% CIs were constructed allowing for arbitrary correlation in the errors within a district. Other covariates include district and year fixed effects; percentage of students who are Asian non-Hispanic, Black non-Hispanic, Hispanic, and other race non-Hispanic; percentage of people within the district boundaries in poverty; percentage of children in the district boundary living in single-parent households; percentage of adults aged 25 years or older within district boundaries with a high school degree, some college, or a 4-year college degree; and real median household income of households with children younger than 18 years within district boundaries.

**Figure 2.**
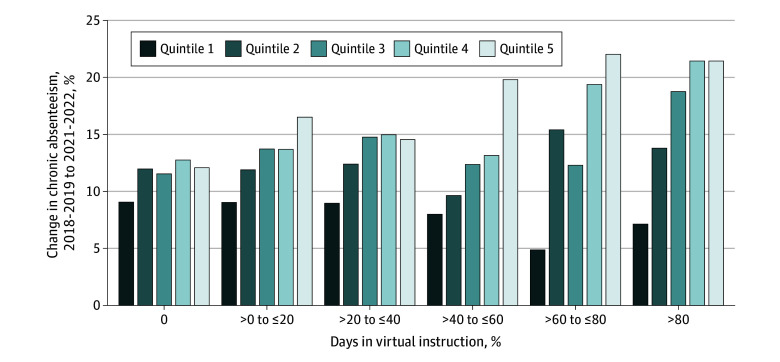
Change in Chronic Absenteeism Rate, 2018-2019 School Year to 2021-2022 School Year, by Percentage of Days in Virtual Instruction and Quintiles of 2018-2019 School Year District-Level Poverty Rates For each of the groups on the x-axis, results are reported for the y-axis by quintile of the 2018-2019 district poverty rate. The numbers are weighted by the mean district size across the 2018-2019 and 2021-2022 school years.

Given the persistent differences in the amount of virtual instruction by poverty rates, we estimated separate regression models for each quintile of the underlying 2018-2019 school year district poverty rate and graphed the student-weighted change in chronic absenteeism between 2018-2019 and 2021-2022 as a function of the fraction of days in virtual education in 2020-2021 and quintiles of the poverty rate (Figure 2). In districts with no days in virtual learning, there was more a modest difference in this time-series change in chronic absenteeism across districts based on poverty. In addition, in districts with the lowest quintile of poverty, the change in chronic absenteeism over time decreased as the percentage of days in virtual leaning increased. In contrast, there was a large increase in the time-series change in chronic absenteeism among the districts with the lowest quintile of poverty as the percentage of virtual days increased.

The results in Figure 2 from the model by quintiles of 2018-2019 district poverty rate are shown in Table 3. In the lowest quintile of poverty, virtual days were associated with a small but statistically significant decrease in chronic absenteeism. The next 2 quintiles have positive but statistically insignificant coefficients on virtual instruction. In the top 2 quintiles, the coefficient of virtual instruction is very large and statistically precise. In the top quartile, having 100% of school days held virtually in the 2020-2021 school year was correlated with a 10.6–percentage point (95% CI, 7.2-14.1 percentage points) increase in chronic absenteeism.

These results are not unique to poverty. In eMethods 3 in Supplement 1, we produce similar patterns of results with 3 other measures of socioeconomic status at the district level: the percentage of adults with a college degree, the median household income, and the percentage of families headed by a single parent. These results are reported in eTables 2, 3, and 4 in Supplement 1, respectively. These results all indicate that there is no statistically significant correlation between virtual instruction and chronic absenteeism in districts with a high percentage of adults with a college degree, high median income, and a low fraction of single-parent families. In contrast, the correlation between percentage of days in virtual instruction and chronic absenteeism is large and statistically significant in districts with the lowest percentage of adults with a college degree, lowest median income, and highest fraction of single-parent families.

## Discussion

The accumulating evidence outlined in the Introduction suggests that virtual learning during the COVID-19 pandemic was detrimental to students’ educational development and mental well-being. Parents, educators, scholars, and the medical community have a few important questions that must be addressed in this area. First, how can these negative consequences be undone? Surveys of both teachers^23^ and school administrators^24^ believe that as we move past the pandemic, virtual instruction will continue to be a major component of K-12 education. A second key question then is how to deliver virtual learning in K-12 learning without these potential negative consequences. Educators and policy makers must be prepared to implement evidence-based policies and practices related to online learning going forward.

### Limitations

This study has some limitations. It does not provide estimates of a causal effect of virtual learning on chronic absenteeism but rather provides suggestive evidence that part of the increase in chronic absenteeism may be due to COVID-19 teaching mode policies.

Chronic absenteeism among public K-12 students has increased considerably in the wake of the COVID-19 pandemic. Much of this increase is not due to the mode of instruction during the 2020-2021 school year, since, as we saw in Figure 2, districts that had 100% of days as in-person instruction also saw increases in absenteeism, although, for this group, there was little difference in changes in chronic absenteeism based on poverty quintiles. The study does not outline the explanation for why this large change occurred. A few possible explanations for this increase could be that 10% to 20% of students are experiencing post–COVID-19 condition symptoms,^20^ there was a corresponding increase in teacher absenteeism that may decrease the attractiveness of attending school,^25,26,27^ the increase in mental health challenges of students,^28,29,30^ an increase in social media use by children,^31^ or a change in parents’ willingness to keep their child out of school in the wake of COVID-19 experiences.^32^

## Conclusions

The results in this cross-sectional study show that virtual learning rates during the 2020-2021 school year and pre–COVID-19 chronic absenteeism rates were both increasing with pre–COVID-19 district poverty rates. The districts that were most at risk for absenteeism were those that relied on virtual learning the most. The results from the regressions show that the virtual learning–chronic absenteeism gradient was largest in these at-risk groups.

## Notes

Supplement 1. **eMethods 1.** Detailed Description of Data **eMethods 2.** Detailed Description of the Statistical Model **eMethods 3.** Supplementary Results **eTable 1.** Fixed-Effect Estimates, Determinants of Percent Chronically Absent, Balanced Panel of Districts, Adding Covariates Where the Coefficient Varies Over Time **eTable 2.** Fixed-Effect Estimates, Determinants of Percent Chronically Absent, Heterogeneity in Results by Percent District Level Adults With a College Degree in 2018/19 School Year, Balanced Panel of Districts **eTable 3.** Fixed-Effect Estimates, Determinants of Percent Chronically Absent, Heterogeneity in Results by Percent District-Level Children That Live in a Single-Parent Household in 2018/19 School Year, Balanced Panel of Districts **eTable 4.** Fixed-Effect Estimates, Determinants of Percent Chronically Absent, Heterogeneity in Results by Median Household Income in 2018/19 School Year, Balanced Panel of Districts
eReferences.

Supplement 2.
Data Sharing Statement
