# Supplementary material for: Virtual Learning in Kindergarten Through Grade 12 During the COVID-19 Pandemic and Chronic Absenteeism
Source: JAMA Netw Open. 2024 Aug 21;7(8):e2429569. doi: 10.1001/jamanetworkopen.2024.29569 (PMC11339657; doi:10.1001/jamanetworkopen.2024.29569)
Supplement: Supplement 1. — eMethods 1. Detailed Description of Data eMethods 2. Detailed Description of the Statistical Model eMethods 3. Supplementary Results eTable 1. Fixed-Effect Estimates, Determinants of Percent Chronically Absent, Balanced Panel of Districts, Adding Covariates Where the Coefficient Varies Over Time eTable 2. Fixed-Effect Estimates, Determinants of Percent Chronically Absent, Heterogeneity in Results by Percent District Level Adults With a College Degree in 2018/19 School Year, Balanced Panel of Districts eTable 3. Fixed-Effect Estimates, Determinants of Percent Chronically Absent, Heterogeneity in Results by Percent District-Level Children That Live in a Single-Parent Household in 2018/19 School Year, Balanced Panel of Districts eTable 4. Fixed-Effect Estimates, Determinants of Percent Chronically Absent, Heterogeneity in Results by Median Household Income in 2018/19 School Year, Balanced Panel of Districts eReferences. [file jamanetwopen-e2429569-s001.pdf]

Supplementary Online Content

Evans WN, Muchnick K, Rosenlund O. Virtual learning in kindergarten through grade 12 during the COVID-19 pandemic and chronic absenteeism. *JAMA Netw Open.* 2024;7(8):e2429569. doi:10.1001/jamanetworkopen.2024.29569

eMethods 1. Detailed Description of Data
eMethods 2. Detailed Description of the Statistical Model
eMethods 3. Supplementary Results
eTable 1. Fixed-Effect Estimates, Determinants of Percent Chronically Absent, Balanced Panel of Districts, Adding Covariates Where the Coefficient Varies Over Time
eTable 2. Fixed-Effect Estimates, Determinants of Percent Chronically Absent, Heterogeneity in Results by Percent District Level Adults With a College Degree in 2018/19 School Year, Balanced Panel of Districts
eTable 3. Fixed-Effect Estimates, Determinants of Percent Chronically Absent, Heterogeneity in Results by Percent District-Level Children That Live in a Single-Parent Household in 2018/19 School Year, Balanced Panel of Districts
eTable 4. Fixed-Effect Estimates, Determinants of Percent Chronically Absent, Heterogeneity in Results by Median Household Income in 2018/19 School Year, Balanced Panel of Districts
eReferences.

This supplementary material has been provided by the authors to give readers additional information about their work.

© 2024 Evans WN et al. *JAMA Network Open.*

eMethods 1. Detailed Description of Data

We construct a district-level dataset that measures chronic absenteeism, student demographics, and characteristics of the population living with district boundaries during the 2018/19 and 2021/22 SYs. Counts of students that are chronically absent by Local Education Agency (LEA, which are mostly school districts) are reported to the National Center for Education Statistics for each school year. This data is available from the ED Data Express web page of the U.S Department of Education.[1] A student is defined as chronically absent if they miss at least 10% of instructional days in a given school year. To calculate absenteeism rates, we divide the number of students that are chronically absent by the total number of students in the LEA, available from the Common Core of Data (CCD), which is reported annually by school and LEA to the National Center for Education Statistics (NCES).[2] The CCD also reports counts of students by race. We use these values to calculate the percent Black non-Hispanic, Hispanic, Asian non-Hispanic, and other-race non-Hispanic, with the fraction white non-Hispanics as the reference group. In the other group, we include American Indian/Alaskan Native, Native Hawaiian/Other Pacific Islander, two or more races, and race not specified.

We collect several control variables from the five-year American Community Survey (ACS) data that have been aggregated to the LEA level. We use data from the 2019 and 2022 five-year ACS for the 2018/19 and 2021/22 SYs, respectively. We include the percent of the district population in poverty and the percent of children under age 18 that live in a single parent household. Additionally, we calculate the percent of adults ages 25 and over that have a high school degree, some college, and a college degree, leaving those with less than a high school degree as a reference group. The median income is collected for households within district

© 2024 Evans WN et al. *JAMA Network Open.*

boundaries and turned into real 2022 values using the Current Price Index for all items, total for the United States.[3]

Finally, we merge information about learning modes during the 2020/21 SY from the COVID-19 School Data Hub.[4] The authors of the web page used data from state agencies to classify the percent of school days at the LEA level that were in-person, hybrid, or virtual during the 2020/21 SY. The web page does not have data from Iowa, Montana, and Oklahoma.

In all, we have four data sets (students counts, demographics within the district, chronic absenteeism rates, and learning modes during COVID) from three different sources (CCD, Census, and Data Hub) and as a result, merging data across these three sources will generate some non-matches. We lose data from the three states not covered from the COVID Data Hub. In general, the mapping of ACS respondents in general does not include charter schools. There are 4,293 LEAs in the CCD that are listed as charter schools and only 30 are in the ACS data. LEAs can combine over time, some LEA's close, and others are started up; therefore, some districts cannot be merged over time. The CCD reports 32 separate LEAs for different components of New York City Public Schools that we aggregate into one district. We also delete 13 LEAs that have greater than 100% chronic absenteeism rate during the 2021/22 SY.

In the CCD, there are 18,609 LEAs in the 2018/19 CCD. Merging data from all sources using the NCES LEA ID we produce an analysis sample of 11,466 LEAs for which we have two-years' worth of data each. We refer to this dataset as our balanced panel of districts. This dataset represents roughly 91% of all K-12 students in the United States in the 2018/19 SY, as well as 93% of students in the 47 states and the District of Columbia in the COVID School Data Hub.

© 2024 Evans WN et al. *JAMA Network Open.*

In the analysis section, we add measures of vaccination rates and COVID case rates at the county level to the basic model. We download population vaccination rate data at the county level as of the end of December, 2021[5] and calculated the fraction of residents that had at least one vaccination by that date. We also calculate average weekly COVID per-person infection rates at the county level from August 1, 2021 through May 31, 2022.[6] These two datasets are merged based on the county where the district is located which is identified from an NCES data file.[7] If the district spans counties, a simple average is taken across counties.

© 2024 Evans WN et al. *JAMA Network Open.*

eMethods 2. Detailed Description of the Statistical Model

Given the panel nature of the data, we estimate a model of the form

(1) $$Y_{it} = X_{it}\beta_1 + Hybrid_{it}\theta_1 + Virtual_{it}\delta_1 + \mu_{1i} + \lambda_{1t} + \varepsilon_{1it}$$

where the key outcome of interest (Y_{it}) is the percent of students that are chronically absent in district i in school year t (t=2018/19 or t=2021/22). We control for a set of time-varying characteristics of residents that live within district boundaries (X_{it}). Given that we have multiple observations per district, we can add a set of mutually exclusive district dummy variables (fixed effects, μ_{1i}) that capture permanent characteristics about districts that do not vary over time such as the urbanicity of the district, the relative size of the district, etc. As much of the differences in outcomes like chronic absenteeism are between district and not within district over time, the set of district fixed effects capture a large fraction of the variation in the outcome of interest. We also control for a year fixed effect (λ_{1t}) that captures factors common to all districts but that vary over time. The final term ε_{1it} is a random error term. In all models, we weight observations by total district school enrollment.

The key covariates are the percent of days which students spent in the previous academic year in either hybrid or virtual instruction, measured by $Hybrid_{it-1}$ and $Virtual_{it-1}$, respectively. As hybrid and virtual instruction were virtually non-existent in K-12 education prior to COVID, the values for $Hybrid_{it-1}$ and $Virtual_{it-1}$ where t-1 is 2017/18 is set to zero. The fixed-effects model is often called a within-group estimator as it attributes to the key coefficients θ_1 and δ_1, the within-panel co-movements in Y and alternate instruction. In this case, districts that moved to hybrid and virtual instruction are “treated” with differing intensities of alternative instruction. Districts that had no hybrid and virtual instruction are a comparison sample. Seeing that they had no change in hybrid and virtual instruction over time, the time-series movements in chronic

© 2024 Evans WN et al. *JAMA Network Open.*

absenteeism represent the secular trend in this outcome that are common to all districts. In this way, our results can be thought of as a difference-in-difference estimate within this two-way fixed-effect framework.

The model above has some limitations. First, because the dependent variable is bounded on the 0-100 interval, the model may predict outside this interval. It is also the case that given the structured nature of the dependent variable, the errors are, by construction, heteroskedastic. It has been noted that these difference-in-difference models have high Type I error rates, primarily because of within-group autocorrelation in errors across observations.[8] The last two problems can be handled easily by using the "clustered" standard error suggested by Liang and Zeger[9] which is a generalization of the procedure of White.[10] This method allows for within-panel correlation in errors and also controls for arbitrary forms of heteroskedasticity while reducing the Type I error rate outlined above.

An alternative to our linear model is to explicitly model the restricted nature of the dependent variable. A standard model would be to estimate a log-odds regression. Define p_{it} to be the fraction of students that are chronically absent in district i in year t with this variable being on the unit interval 0 to 1. The log-odds version of equation (1) would then be

$$\text{(2)} \qquad \ln[p_{it}/(1-p_{it})] = X_{it}\beta_2 + Hybrid_{it}\theta_2 + Virtual_{it}\delta_2 + \mu_{2i} + \lambda_{2t} + \varepsilon_{2it}$$

where the terms are define similarly to above. The predicted value from this regression would then map back into a predicted p_{it} that would fall on the unit interval. A shortcoming of this model is that the dependent variable is not defined when p_{it} equals 1 or 0. In our case, p_{it} equals 0 in a dozen cases, therefore, we would either lose those observations or use an ad hoc procedure so as not to not lose that data. In the next section, we summarize estimates for equation (2)

© 2024 Evans WN et al. *JAMA Network Open.*

where we set p_{it}=0.0001 for the observations where the true value is zero to generate comparable estimates.

A competitor model to (1) is to estimate the equation assuming that the district effects (μ_{1i}) are random effects which is a more efficient model than fixed effects. We did not use this model because the random effects model assumes that that the permanent differences across districts are random draws and hence uncorrelated with underlying control variables that would be included in X_{it}. Under the null hypothesis that the effects are indeed random, fixed effects should provide statistically similar but less efficient estimates. If the random effects are correlated with the underlying X's the random effects will be inconsistent. We can test this hypothesis with a Hausman test.[11] We summarize those results in the next section and identify why we use the fixed-effects model.

In Table 3, we estimate models that allow the coefficient on virtual and hybrid education to vary based on district characteristics in the 2018/19 school year. In these models, we take a district characteristic like poverty rates in the 2018/19 SY and place districts into quintiles. We then estimate separate models for each quintile.

© 2024 Evans WN et al. *JAMA Network Open.*

eMethods 3. Supplementary Results

The COVID-19 pandemic disrupted many aspects of daily life and health, and economic consequences of the pandemic were not uniform in the population.[12,13,14] The coefficient on virtual days could potentially be capturing the fact that there was a fundamental change in the impact of the control variables on chronic absenteeism brought about by the events of COVID rather than through the method of schooling. To test this, we allow the coefficients to vary over time. We do this by adding an interaction between the variables in our X vector with a dummy variable for the 2021/22 SY. We first add one interaction for each of the 10 covariates one at a time, then add all 10 interactions at once. These results are reported in eTable 1 below.

In the first row of the table, we reproduce the coefficients for % days hybrid and % days virtual from the basic model found in Table 2 in the main body of the paper. In the next 10 rows, we add an interaction for one variable and the year 21/22 dummy. In the last row, we report the model when all interactions are added. When interactions are added one at a time, the coefficients changes some but the results are always statistically significant. The largest change occurs when we add the interaction with percent Hispanic and the year dummy. We do not believe our results are being driven by a fundamental change in the relationship between the fraction Hispanic students and chronic absenteeism over time, as we run a separate model that deletes any district with greater than or equal to 5% Hispanic in both years of the panel. This leaves 4003 districts and 8,006 observations in a regression. In this much smaller sample, the coefficient (95% CI) on % days in virtual instruction is 0.096 (0.061, 0.131). If we reduce this to districts with <1% Hispanics in both years, we have 1,048 districts and the coefficient (95% CI) on % days in virtual instruction is now 0.117 (0.043, 0.192).

© 2024 Evans WN et al. *JAMA Network Open.*

As we noted above, an alternative model is to explicitly model the limited nature of the dependent variable. One way to do this is through a log-odds regression where the dependent variable is the natural log of the odds ratio of the fraction chronically absent (p_{it}) as in equation (2). In our sample, we do have a dozen observations where p_{it} =0 and the dependent variable is undefined. To keep these observations in the regression, we redefine p_{it} =0.0001 for those observations and re-estimate the model. In this case, we obtain a coefficient (θ_2) on virtual days of 0.0023 (95% CI of 0.0012, 0.0013). It can be shown that the gradient dp_{it}/d(virtual days)= $\theta_2 p_{it}(1-p_{it})$. Letting p_{it} be the mean of the sample in 2021/22 SY (0.294) we obtain dp_{it}/d(virtual days) = 0.00047 which means that schools with 100% virtual instruction in the 2020/21 SY experienced an increase in chronic absenteeism of 0.047 or 4.7 percentage points, which is similar to the results in Table 2.

In Table 3 in the main body of the paper, we report the heterogeneity of results based on an indicator of students at-risk for chronic absenteeism: poverty. In this section, we repeat the results from Table 3 with three additional measures: the fraction of adults aged 25 and above with a college degree, the fraction of households with single parents, and the real median household income for households with their own children under 18. These results are reported in eTables 2, 3, and 4 respectively, with the structure of the table mirroring that of Table 3.

eTable 2 contains heterogeneity in the results using the percent of parents with a college degree. The basic patterns are similar to Table 3 in that in rows (1) through (3), we see that virtual instruction was used more frequently in the districts with the lowest percent of college-educated adults, and in row (4), we see that this same group has the highest chronic absenteeism rates prior to COVID. In row (5), in the top quintile group, there is a small and statistically imprecise impact of virtual days on absenteeism. In the four lowest quintiles, all effects are large

© 2024 Evans WN et al. *JAMA Network Open.*

and statistically significant, and the results are increasing in size as the fraction of adults with college degrees declines. The results in the lowest quintile suggest that 100% of days in virtual learning during the 2020/21 SY would have produced a rise in chronic absenteeism rate of 14.6 percentage points (95% CI of 11.1 to 18.1).

The results when heterogeneity is based on percent single parents (eTable 3) and median household income (eTable 4) produce similar patterns. In rows (1) through (3) of the tables, we see that virtual instruction is used most in the highest quintile of single parents and the lowest quintile of median household. These are the same groups that have the highest pre-COVID chronic absenteeism rates (row (4) of the tables). In row (5) of the tables, in the lowest quintile of single parents and the highest quintile of median family income, the coefficient on days in virtual instruction is statistically insignificant. However, in the highest quintile of single parents, students that spent 100% of days in virtual instruction had an 8.4 percentage point higher chronic absenteeism (95% CI of 4.6 to 12.3). Likewise, in the lowest quintile of median family income, those that spent 100% of days in virtual income experienced a 9-percentage point increase in chronic absenteeism (95% CI of 5.1 to 12.9).

The results in Table 3 and eTables 2 through 4 demonstrate definitively that the use of virtual instruction in the 2020/21 SY was related to underlying characteristics of the school district. In row (3) of these four tables, we see that virtual instruction was decidedly higher in districts with high rates of poverty and single parenthood and lower levels of income and college educated adults. In row (4) of those tables, we also see that the same districts that had these characteristics also had much higher levels of underlying chronic absenteeism prior to COVID. This suggests that the primary assumption of the more efficient random effects model may be in error. To verify this point, we estimate a version of equation (1) under random and fixed effects,

not using district weights and not clustering the standard errors at the district level. The coefficient (95% confidence interval) [standard error] on the coefficient for virtual days in the random effects model is 0.098 (0.091, 0.105) [0.0035] while the estimate from the fixed effects model is 0.077 (0.069, 0.084) [0.0039]. As expected, the random effects model is more efficient but a Hausman test easily rejects that null hypothesis of equality of the two coefficients (p-value < 0.001) which suggests the assumptions of the random effects model is incorrect. It is also the case that the standard error in this fixed-effects model is incredibly small which is consistent with the notion that fixed-effects models have high Type I error rates[8] so the clustering procedure of Liang and Zeger is necessary.[9]

© 2024 Evans WN et al. *JAMA Network Open.*

eTable 1. Fixed-Effect Estimates, Determinants of Percent Chronically Absent, Balanced Panel of Districts, Adding Covariates Where the Coefficient Varies Over Time

	Coefficient (95% Confidence Interval)	
Change in specification	% days hybrid	% days virtual
Baseline specification	-0.003 (-0.019, 0.013)	0.069 (0.048, 0.089)
Add interaction of a 2021/22 dummy x		
% students Black, non-Hispanic	-0.003 (-0.019, 0.013)	0.068 (0.047, 0.088)
% students Asian, non-Hispanic	-0.001 (-0.019, 0.017)	0.080 (0.057, 0.102)
% students other race, non-Hispanic	-0.003 (-0.019, 0.013)	0.069 (0.049, 0.090)
% students Hispanic	0.014 (0.001, 0.027)	0.038 (0.018, 0.058)
% district population in poverty	0.004 (-0.010, 0.017)	0.058 (0.038, 0.077)
% adults in district aged 25+ with a high school degree	-0.002 (-0.018, 0.015)	0.074 (0.053, 0.095)
% adults in district aged 25+ with some college	0.001 (-0.016, 0.019)	0.073 (0.052, 0.093)
% adults in district aged 25+ with a 4-year college degree	0.006 (-0.010, 0.023)	0.073 (0.053, 0.093)
% children in district that live in a single-parent HH	0.002 (-0.019, 0.016)	0.056 (0.037, 0.076)
Median HH income among HH with children < 18, real 2022 $	0.009 (-0.004, 0.022)	0.070 (0.051, 0.089)
Add all interactions	0.024 (0.012, 0.036)	0.029 (0.009, 0.050)

Observations are weighted by total enrollment in the district. Confidence intervals were constructed allowing for arbitrary correlation in the errors within a district. Other covariates include district and year fixed effects, the percent of students that are black non-Hispanic, Asian non-Hispanic, other race non-Hispanic, and Hispanic, the percent of people within the district boundaries in poverty, the percent of children in the district boundary living in single-parent households, percent of adults aged 25 and over within district boundaries with a high school degree, some college or a 4-year college degree, and real median household income of households with children under 18 within district boundaries.

© 2024 Evans WN et al. *JAMA Network Open.*

eTable 2. Fixed-Effect Estimates, Determinants of Percent Chronically Absent, Heterogeneity in Results by Percent District Level Adults With a College Degree in 2018/19 School Year, Balanced Panel of Districts

		Quintile of district characteristic % of adults aged 25+ with a college degree in 2018/19 SY				
Row/Definition	Variable	1st	2nd	3rd	4th	5th
% of days in learning mode SY 2020/21						
(1)	In person	41.7	43.4	47.9	41.5	31.6
(2)	Hybrid	26.0	29.6	27.9	31.5	42.1
(3)	Virtual	32.3	27.0	24.2	27.0	26.3
% Chronically absent during 2018/19 SY						
(4)		18.4	17.3	17.5	16.8	13.2
Parameter Estimates (95% Confidence Interval) on days in mode						
(5)	% days hybrid	-0.009	-0.009	0.012	0.011	-0.002
		(-0.037, 0.020)	(-0.036, 0.017)	(-0.017, 0.040)	(-0.016, 0.037)	(-0.027, 0.023)
(6)	% days virtual	0.146	0.108	0.092	0.064	0.009
		(0.111, 0.181)	(0.061, 0.154)	(0.054, 0.129)	(0.025, 0.103)	(-0.032, 0.051)

Observations are weighted by total enrollment in the district. Confidence intervals constructed allowing for arbitrary correlation in the errors within a district. Other covariates include district and year fixed effects, the percent of students that are Black non-Hispanic, Asian non-Hispanic, other race non-Hispanic, and Hispanic, the percent of people within the district boundaries in poverty, the percent of children in the district boundary living in single-parent households, percent of adults aged 25 and over within district boundaries with a high school degree, some college or a 4-year college degree, and real median household income of households with children under 18 within district boundaries.

© 2024 Evans WN et al. *JAMA Network Open.*

eTable 3. Fixed-Effect Estimates, Determinants of Percent Chronically Absent, Heterogeneity in Results by Percent District-Level Children That Live in a Single-Parent Household in 2018/19 School Year, Balanced Panel of Districts

		Quintile of district characteristic nu % of children in district that live in a single-parent household, 2018/19 SY				
Row/Definition	Variable	1st	2nd	3rd	4th	5th
% of days in mode SY 2020/21						
(1)	In person	42.3	39.9	36.6	41.3	37.3
(2)	Hybrid	41.9	38.1	38.0	30.2	27.5
(3)	Virtual	15.8	22.0	25.4	28.6	35.3
% Chronically absent during 2018/19 SY						
(4)		8.7	11.6	14.0	18.2	21.3
Regression Parameter Estimates (95% Confidence Interval) on days in mode						
(5)	% days hybrid	0.007	-0.001	-0.002	-0.007	0.013
		(-0.018, 0.032)	(-0.024, 0.022)	(-0.025, 0.021)	(-0.034, 0.020)	(-0.016, 0.043)
(6)	% days virtual	-0.030	0.008	0.074	0.077	0.084
		(-0.068, 0.007)	(-0.030, 0.046)	(0.038, 0.111)	(0.044, 0.110)	(0.046, 0.123)

Observations are weighted by total enrollment in the district. Confidence intervals were constructed allowing for arbitrary correlation in the errors within a district. Other covariates include district and year fixed effects, the percent of students that are Black non-Hispanic, Asian non-Hispanic, other race non-Hispanic, and Hispanic, the percent of people within the district boundaries in poverty, the percent of children in the district boundary living in single-parent households, percent of adults aged 25 and over within district boundaries with a high school degree, some college or a 4-year college degree, and real median household income of households with children under 18 within district boundaries.

© 2024 Evans WN et al. *JAMA Network Open.*

eTable 4. Fixed-Effect Estimates, Determinants of Percent Chronically Absent, Heterogeneity in Results by Median Household Income in 2018/19 School Year, Balanced Panel of Districts

		Quintile of district characteristic				
Row/definition	Variable	1st	2nd	3rd	4th	5th
% of days in learning mode in SY 2020/21						
(1)	In person	38.1	39.8	50.5	37.6	32.4
(2)	Hybrid	26.0	31.7	28.7	37.0	43.6
(3)	Virtual	35.9	28.5	20.8	25.4	24.0
Chronically absent during 2018/19 SY						
(4)		21.1	19.3	15.7	14.0	9.8
Parameter Estimates (95% Confidence Interval) on days in mode						
(5)	% days hybrid	0.009	-0.001	0.002	0.006	-0.003
		(-0.021, 0.040)	(-0.032, 0.031)	(-0.020, 0.024)	(-0.019, 0.033)	(-0.024, 0.018)
(6)	% days virtual	0.090	0.111	0.044	0.065	-0.007
		(0.051, 0.129)	(0.074, 0.148)	(0.004, 0.084)	(0.022, 0.109)	(-0.038, 0.024)

Observations are weighted by total enrollment in the district. Confidence intervals were constructed allowing for arbitrary correlation in the errors within a district. Other covariates include district and year fixed effects, the percent of students that are Black non-Hispanic, Asian non-Hispanic, other race non-Hispanic, and Hispanic, the percent of people within the district boundaries in poverty, the percent of children in the district boundary living in single-parent households, percent of adults aged 25 and over within district boundaries with a high school degree, some college or a 4-year college degree, and real median household income of households with children under 18 within district boundaries.

© 2024 Evans WN et al. *JAMA Network Open.*

eReferences

1. https://eddataexpress.ed.gov/download/data-library. Accessed February 26, 2024.

2. https://nces.ed.gov/ccd/files.asp. Accessed February 26, 2024.

3. https://fred.stlouisfed.org/series/USACPALTT01CTGYM. Accessed February 26, 2024.

4. https://www.covidschooldatahub.com/. Accessed February 26, 2024.

5. https://data.cdc.gov/Vaccinations/COVID-19-Vaccinations-in-the-United-States-County/8xkx-amqh/about_data. Accessed February 26, 2024.

6. https://data.cdc.gov/Public-Health-Surveillance/Weekly-COVID-19-County-Level-of-Community-Transmis/jgk8-6dpn/about_data. Accessed February 26, 2024.

7. https://nces.ed.gov/programs/edge/geographic/relationshipfiles

8. Bertrand, M., Duflo, E. and Mullainathan, S. (2004). How much should we trust differences-in-differences estimates? *The Quarterly Journal of Economics*, *119*(1), 249-275.

9. Liang, K.Y. and Zeger, S.L. (1986). Longitudinal data analysis using generalized linear models. *Biometrika*, *73*(1), 13-22.

10. White, H. (1980). A heteroskedasticity-consistent covariance matrix estimator and a direct test for heteroskedasticity. *Econometrica*, *48(4)*, 817-838.

11. Hausman, J.A. (1978). Specification tests in econometrics. *Econometrica 46(6)*, 1251-1271.

12. Finkelstein, A., Kocks, G., Polyakova, M., & Udalova, V. (2022). Heterogeneity in Damages from a Pandemic (No. w30658). National Bureau of Economic Research.

13. Alsan, M., Chandra, A., & Simon, K. (2021). The great unequalizer: initial health effects of COVID-19 in the United States. *Journal of Economic Perspectives*, *35*(3), 25-46.

14. Cronin, C. J., & Evans, W. N. (2021). Excess mortality from COVID and non-COVID causes in minority populations. *Proceedings of the National Academy of Sciences*, *118*(39), e2101386118.

© 2024 Evans WN et al. *JAMA Network Open.*
