# Supplementary material for: Virtual Learning in Kindergarten Through Grade 12 During the COVID-19 Pandemic and Chronic Absenteeism
Source: JAMA Netw Open. 2024 Aug 21;7(8):e2429569. doi: 10.1001/jamanetworkopen.2024.29569 (PMC11339657; doi:10.1001/jamanetworkopen.2024.29569)
Supplement: Supplement 2. — Data Sharing Statement [file jamanetwopen-e2429569-s002.pdf]

Data Sharing Statement

Evans. Virtual Learning in Kindergarten Through Grade 12 During the COVID-19 Pandemic and Chronic Absenteeism. *JAMA Netw Open*. Published August 21, 2024. doi:10.1001/jamanetworkopen.2024.29569

Data
Data available: Yes
Data types: Participant data with identifiers, Data (not involving human participants), Data dictionary
How to access data: openicpsr.org
When available: With publication

Supporting Documents
Document types: Statistical/analytic code
How to access documents: openicpsr.org
When available: With publication

Additional Information
Who can access the data: Anyone requesting the data
Types of analyses: For any purpose
Mechanisms of data availability: Without investigator support
